# Supplementary material for: Hydralazine targets cAMP-dependent protein kinase leading to sirtuin1/5 activation and lifespan extension in C. elegans
Source: Nat Commun. 2019 Oct 28;10:4905. doi: 10.1038/s41467-019-12425-w (PMC6817882; doi:10.1038/s41467-019-12425-w)
Supplement: Supplementary file 1 — Supplementary Information [file 41467_2019_12425_MOESM1_ESM.docx]

**Supplementary Information:**

**Hydralazine targets cAMP-dependent kinase leading to sirtuin1/5 activation and lifespan extension in *C. elegans***

Dehghan et al.

Table of contents

I. Supplementary Figures

Supplementary Figure 1: Supplementary Figure for Figure 2.

Supplementary Figure 2: Supplementary Figure for Figure 3.

Supplementary Figure 3: Supplementary Figure 1 for Figure 4.

Supplementary Figure 4: Supplementary Figure 2 for Figure 5.

Supplementary Figure 5: Supplementary Figure for Figure 6.

Supplementary Figure 6: Uncropped scans for Figure 1c.

Supplementary Figure 7: Uncropped scans for Figure 2b, 2d, 2e, and 2h.

Supplementary Figure 8: Uncropped scans for Figure 5b and 5e.

II. Supplementary Tables

Supplementary Data 1: Supplementary Data for Figure 4.

Supplementary Table 2: Supplementary Table for Figure 2

**Supplementary Figure 1. Investigating the role of sirtuins in hydralazine-mediated mitochondrial activation. (a)** Gene expression analysis of *SIRT1*-*SIRT7* in SH-SY5Y cells treated with hydralazine for 8 h (*n = 3*). **(b-c)** Western blot confirmation of SIRT1 and SIRT5 knockdown in SH-SY5Y cells(SIRT1 *n = 3,* SIRT5 *n = 2*). **(d)** TMRE signal quantification of mitochondrial membrane potential indicating inhibitory effects of SIRT1 antagonists, NAM and sirtinol, on hydralazine induced mitochondrial activation (*n = 2*)*.* **(e)** Measurement of mitochondrial membrane potential showing activation of mitochondria in control and HIF1A knockdown SH-SY5Y cells treated with hydralazine (20 µM) for 72 h (*n = 2*). **(f)** Measurement of survival rate in *C. elegans* under rotenone stress indicates a HIF1A-independent hydralazine mode of action (*n = 2*). *p ≤ 0.05, **p ≤ 0.05, two-tailed Student's *t* test, mean ± SD.

**Supplementary Figure 2. Correlation between mitochondrial activation and prolongevity effects of hydralazine.** Lifespan analysis of *C. elegans* with impaired mitochondrial ETC complex I (*gas-1* mutants) treated with hydralazine indicating a necessary role for mitochondrial activation in prolongevity properties of hydralazine.

**Supplementary Figure 3. Metabolomics analysis of hydralazine treated *C. elegans.*** **(a)** Mass spectral analysis of the TCA cycle metabolites in *C. elegans* shows an overall reduction in mitochondrial metabolism flux as a result of high-glucose diet which can be reversed by hydralazine treatment (4 days). **(b)** Cell viability assay demonstrated a SIRT1 and SIRT5-dependent recovery of AP cells with hydralazine treatment (5 µM). **(c)** Reduced protein abundances of SIRT5 and TFAM in AP cells was restored with hydralazine treatment as shown with Western blot analysis*.* **(d)** DAVID (the database for annotation, visualization and integrated discovery) analysis of label free global comparative proteomics data from wild-type *C. elegans* treated with hydralazine (100 µM), under rotenone stress condition, showing activation of mitochondria. **p ≤0.05, **p ≤ 0.01* two-tailed Student's *t*-test*, n = 3,* mean ± SD*.*

**Supplementary Figure 4.** **Identification of hydralazine binding target.** **(a)** *In silico* simulation showing interaction of hydralazine with catalytic subunit of PKA. **(b)** Mass spectral analysis of cAMP in the SH-SY5Y cells shows that cAMP does not change with hydralazine treatment (*n = 5,* mean ± SD). **(c)** Western blot analysis indicating elimination of catalytic subunit of PKA in HEK293 cells. **(d)** Measurement of Δψm demonstrating a KIN-1-dependent elevation in mitochondrial activity in *C. elegans* treated for 3 days (*n = 4*, mean ± SEM). **(e)** Western blot analysis demonstrating a PKA-dependent upregulation of SIRT1, NRF2 and HMOX1 in HEK293 cells treated with 10 µM hydralazine for 24 h (*n= 3*, mean ± SEM). *p ≤ 0.05, **p < 0.01 two-tailed Student's *t*-test.

**Supplementary Figure 5. The effect of dose on hydralazine-mediated mitochondrial respiration. (a)** Oxygen consumption rate in the C2C12 cells treated with hydralazine for 3 days measured by Seahorse analysis. Data representing normalized OCR to the protein amount (*n = 4*). **(b)** Fluorescent photomicrographs of transgenic *C. elegans* expressing [*hsp-6::GFP*] reporter does not support activation of UPRmt with hydralazine treatment (*n = 2*). **(c)** Western blot analysis demonstrating the effect of SIRT1 activation on the abundances of NRF2 in C2C12 cells treated for 48 h with hydralazine (*n = 3*). *p ≤ 0.05, **p < 0.01 two-tailed Student's *t*-test, mean ± SD.


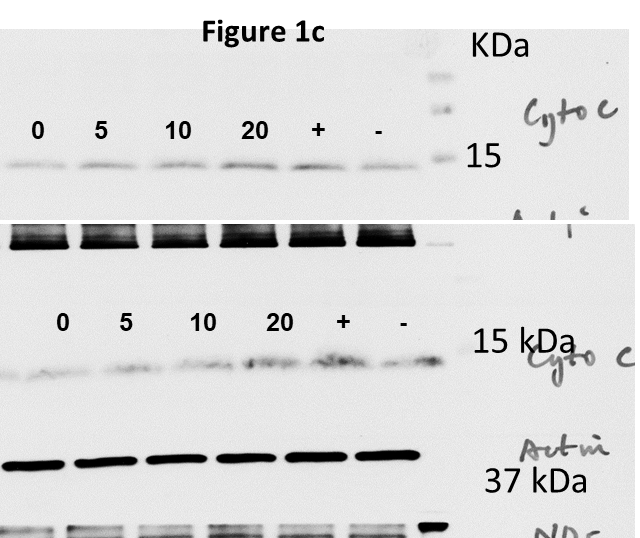


**Supplementary Figure 6: uncropped scans for Figure 1c.**


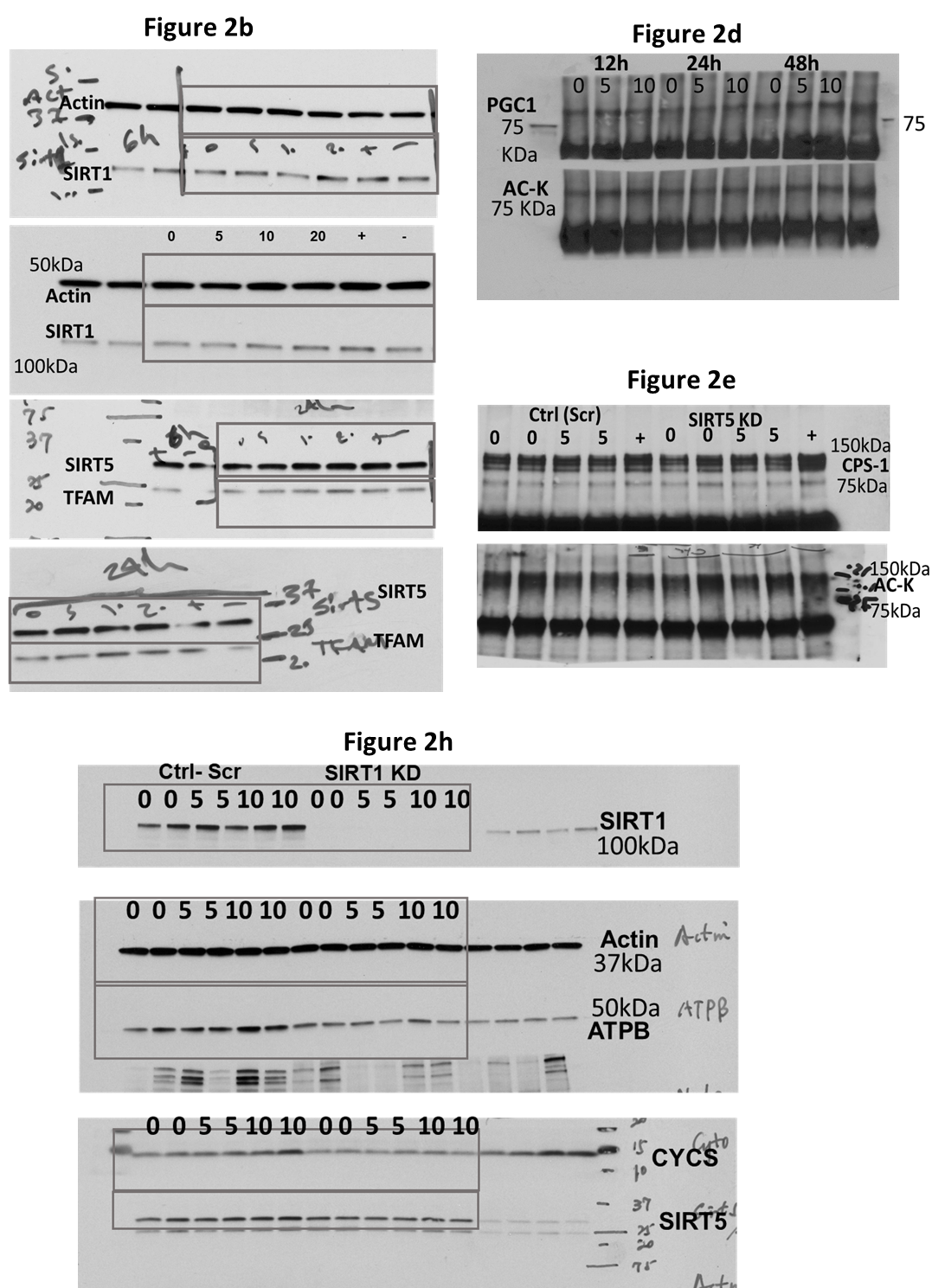


**Supplementary Figure 7: uncropped scans for Figure 2b, 2d, 2e, and 2h**


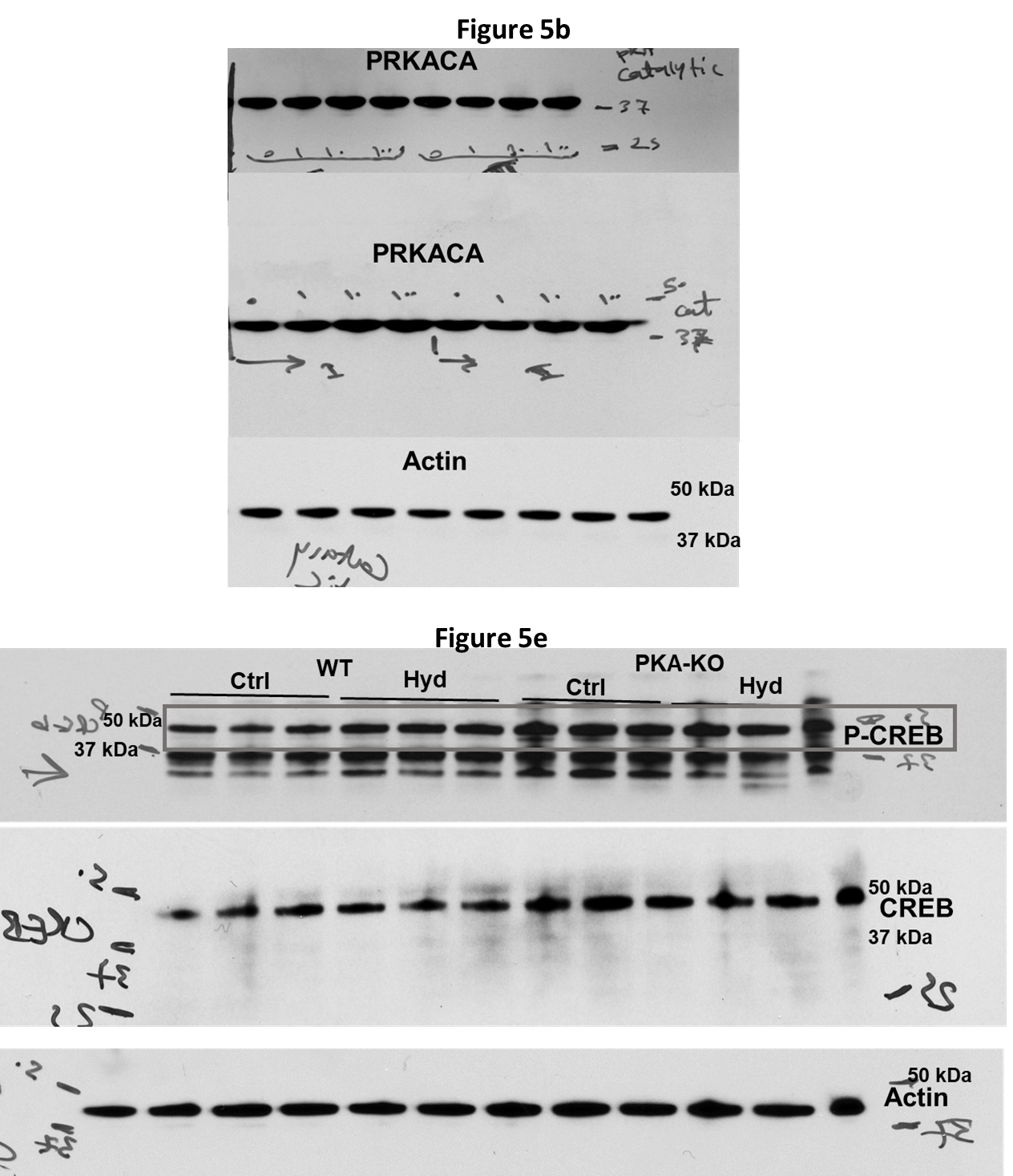


**Supplementary Figure 8: uncropped scans for Figure 5b and 5e.**

**Supplementary Table 1.** *C. elegans* lifespan statistics

|  | N2- Ctrl | 15.0±0.00 | - | - | 65 (1) |
| --- | --- | --- | --- | --- | --- |
| **3 (G)** | N2- 100 µM Hyd | 18.0±0.00 | +20.00 | <0.0001 | 68 (1) |
|  | N2- 0.1 µM AA | 15.0±0.00 | - | - | 117 (2) |
|  | N2- 0.1 µM AA+ 100 µM Hyd | 15.5±0.70 | +3.33 | 0.1064 | 118 (2) |
|  | *sir-2.1*- Ctrl | 14.5±0.70 | - | - | 148 (2) |
| **3 (H)** | *sir-2.1*- 100 µM Hyd | 15.0±0.00 | +03.48 | 0.0761 | 128 (2) |
|  | *mev-1*- Ctrl | 13.5±0.70 | - | - | 156 (2) |
|  | *mev-1*- 100 µM Hyd | 13.5±0.70 | 0 | 0.7463 | 171 (2) |
| **2S (A)** | *gas1*-Ctrl | 13.0±1.40 | - | - | 145 (2) |
|  | *gas1*-100 µM Hyd | 13.0±1.40 | 0 | 0.6413 | 149 (2) |
|  | N2- Ctrl | 17.0±0.00 | - | - | 152 (2) |
| **4(A)** | N2- 100 µM Hyd | 20.5±0.70 | +20.58 | <0.0001 | 144 (2) |
|  | N2- 50mM Glu | 14.0±0.00 | -17.67 | <0.0001 | 163 (2) |
|  | N2- 50mM Glu + 100 µM Hyd | 16.0±0.00 | +14.28 | <0.0001 | 159 (2) |
|  | *rrf-3/fem-1* Ctrl RNAi-0 | 15.0±0.00 | - | - | 363(1) |
| **5 (I)** | *rrf-3/fem-1* Ctrl RNAi-100 µM Hyd | 19.0±0.00 | +26.66 | <0.0001 | 343(1) |
|  | *rrf-3/fem-1* kin-1 RNAi-0 | 13.0±0.00 | - | - | 302(2) |
|  | *rrf-3/fem-1* kin-1 RNAi-100 µM Hyd | 13.0±0.00 | 0 | 0.0064 | 282(2) |

**Supplementary Table 2.** A list of primer sequences used for quantitative real-time PCR analysis
