# Supplementary material for: Hydralazine targets cAMP-dependent protein kinase leading to sirtuin1/5 activation and lifespan extension in C. elegans
Source: Nat Commun. 2019 Oct 28;10:4905. doi: 10.1038/s41467-019-12425-w (PMC6817882; doi:10.1038/s41467-019-12425-w)
Supplement: Supplementary file 3 — Description of Additional Supplementary Files [file 41467_2019_12425_MOESM3_ESM.docx]

**Title:** Supplementary Data 1

**Description:** Relative mass spectral quantification of metabolites extracted from control and hydralazine treated wild-type C. elegans (100 µM, 4 days) grown in absence or presence of a high glucose concentration.
